# The effect of group integrative reminiscence therapy on mental health among older women living in Iranian nursing homes

**DOI:** 10.1002/nop2.101

**Published:** 2017-10-05

**Authors:** Motahareh Musavi, Sara Mohammadian, Bahar Mohammadinezhad

**Affiliations:** ^1^ Department of Nursing and Midwifery Dezful University of Medical Sciences Dezful Iran

**Keywords:** group therapy, integrative reminiscence, mental health, older people

## Abstract

**Aim:**

The present study aimed to determine the impact of group integrative reminiscence therapy on mental health of older women living in Iranian nursing home in 2016.

**Design:**

A quasi‐experimental design pre‐ and post‐test was used in the present study. Purposive sampling was used to recruit participants who met the study criteria.

**Method:**

The statistical population included 60 older women living in nursing home, among whom 46 having the inclusion criteria were selected as the sample. After completing the Goldberg's General Health Questionnaire, the older people were divided into intervention (*N *=* *23) and control (*N *=* *23) group by using block randomization method. The intervention groups were trained by integrative reminiscence therapy for 60–90 min 10 sessions, twice a week. Descriptive statistics and ANOVA were used for data analysis by SPSS software.

**Results:**

The results indicated that group integrative reminiscence therapy significantly affected general mental health and physical dimensions, anxiety and insomnia, social functions and depression.

## 1 INTRODUCTION

According to WHO definition, the age more than 60 is regarded as an old age. According WHO's classification, older people are divided into three groups including the young‐old (60–74), old (75–90) and old‐old (over 90) (Kaplan & Sadok, 2007; Knight, 2004). Old age is a normal process of growth and development in human leading physiological, mental and social changes among older people. These changes make body susceptible to diseases with several clinical demonstrations.

Based on the report of world population in 2005, 7.4% of the total population are the older people over 65 years (Brunner & Suddarth, 2008) and it will reach over 10.4% by 2025 (Bailey, 2009; Bloom, Canning, & Fink, 2010). According to the latest census report, more than 5 million seniors are living in Iran now (Amini, Ingman, & Sahaf,^2013^) and it is predicted that more than 26 million seniors (over 60 years old)) (Kiani, 2010; Nejati et al., 2008; WHO, 2009) will live in Iran by 2050 (Sheykhi, 2006). Currently, more than half of the world older populations are women, the majority of whom are living in developing countries (WHO, 1998).

Obviously, an increasing number of older people results in increasing the number of nursing homes (Van & Weyden, 1999). In Iran, the population of older people living in nursing homes is 10–12 thousands, among whom almost 0.24% are institutionalized in day care centres or 24‐hour facilities. Thus, more than 99% of older people are informally receiving care and the majority of the seniors’ health care is based on informal care (Amini et al., 2013). Living in these centres may create several problems such as the prevalence of mental health disorders (Boorsma, Joling, Dussel, Ribbe, & Frijters, 2012). In addition, the diversity and the amount of stress among older people in relation to their surrounding environment such as losing jobs, social position and close relatives have emphasized the mental health of older people (Sullivan‐Marx, 2013). Mental disorders are regarded as one of the most important elements of global burden diseases and it is estimated that, the ratio of mental and neurological disorders to global burden diseases would increase up to 50% by 2020, reaching from the current 10.5% to 15% of global burden of disease (Olfson, 2002).

During older people, the sense of loneliness develops due to weak social relations, which can influence lifestyle and the amount of life satisfaction in addition to physical and psychological health (Henrich & Gullone, 2006; Snodown, 2001). Lauder, Mummery, and Sharkey (2006) indicated that 35%–48% of older people over 65 years in the USA were suffering from loneliness. Mohammadi et al. (2005), in their study, emphasized the prevalence of mental disorders among women was 1.44%, which is higher than that of men (1.35%). Among the reasons, stressor due to unemployment, lack of income, limitations of social relations and monotonous life were emphasized.

According to Sadeghi and Kazemi (2004), 16.8% and 14.7% of older people are suffering from minor and major depression, respectively.

Older people can enjoy a happy and productive life by adopting an appropriate planning and were accepted as an integrated part of the society. Among these plans, we can refer to psychotherapy methods (Woods, Spector, Jones, Orell, & Davies, 2008). The psychotherapy in older people aims to provide support, reduce anxiety and increase socialization (Sadock & Sadock, 2007). Group therapy is a form of psychotherapy where all members of the group are familiarized with other people's experiences and accordingly experience new behaviours, cope with stress and reconcile with decreasing resources of life through peer emotional support from peers, (Hsu & Wang, 2009). Participation in arranged reminiscence groups is regarded as a type of effective and almost without any unfavorable group therapy. Watt and Wong (1991) identified six types of reminiscence including integrative, instrumental, transmissivity, narrative, escapist and obsessive, among which integrative and instrumental reminiscence are more beneficial to successful ageing.

Integrative reminiscence focuses on accepting one's past, looking towards one's past life as a valuable and satisfying period, finding meaning in life, narrowing the gap between reality and the ideal, accepting past negative events and recognizing a continual pattern between past and present (Mohammadzaddeh, Dolatshahi, & Mohammadkhani, 2011). According to Watt and Cappeliez (1991), integrative reminiscence therapy aims at a constructive re‐appraisal of interpretations and emotions towards past self‐defining events.

American Nurses Association supports reminiscence as a standard nursing intervention and believes that it can be used as an intervention for older people in nursing homes (Wu, 2011). The first approach works best with older adults who have relatively good mental health and involve the simple recollection of positive autobiographical events to foster positive emotions. However, reminiscence on the life review focuses on both positive and negative memories, encompassing the entire life span. This intervention is used to perform a critical analysis of one's life history and achieve ego integrity (Haber, 2006).

Wu (2011), in his study, demonstrated an immediate impact on the symptoms of depression, life satisfaction and confidence. Chiang et al. (2009) believe that the reminiscence therapy helps to prevent mental disorders. Karimi et al. (2010) emphasized that integrative reminiscence plays a significant role on reducing the symptoms of depression among older people. In another study, Majzoobi, Momeni, Amani, and Hojjat (2013) indicated that the intervention was more effective on happiness although it failed to affect the life quality significantly. In geriatric nursing, integrative reminiscence group therapy is considered as a low‐cost, easy and independent intervention, having some positive impacts, without any harmful side effect. However, this kind of intervention is not common in Iran and little is known about its effectiveness. Increasing older population, the need for psychotherapy interventions and research costs are regarded as the significance of conducting such studies.

Regarding a lack of effective, adapted and studied psychological treatment on the problems related to older people in Iran and confirming the effectiveness of this medical intervention in the previous studies, the present study aimed to determine the impact of integrative reminiscence group therapy on mental health of older women living in Masume nursing home in 2016.

## 2 METHOD

The present study is a quasi‐experimental study based on pre‐ and post‐test. The field study started after and the validity and reliability of the instruments were confirmed and ethical approval letter was obtained from the ethics committee of the university. Then, the related centres were observed and the necessary coordination was made by the related authorities in the centres.

Some inclusion criteria were considered for selecting the subjects such as 60 years and older, awareness of time, space and objects, an inclination towards participation in the study, staying at nursing home for at least 6 months and not receiving any treatment which causes any intervention in mental ability, memory or thoughts. Some exclusion criteria like acute physical and mental disorders, cognitive disorders and previous history of attending in reminiscence sessions and simultaneous participation in other psychotherapy sessions were taken into consideration in the present study.

The statistical population included 60 older people in nursing homes, among whom 46 having the required criteria were selected as the main subjects. Then, they completed the consent form to be involved in the study. It is worth noting that the mental health of the participants was investigated by a psychiatrist and a consultant psychologist to help identify problems and mental disorders of older people. In the next stage, older people were asked to fill the demographic questionnaire and Goldberg mental health questionnaire and they were coded through personal interview to explain and respond the questionnaire by the interviewer for all samples.

Then, the older people were equally divided into intervention and control group by using block randomization method. According to the standard number of people in group therapy and better treatment effects (Sharf, 2007), the intervention group were divided into two groups of 10 and 11 people. In this method, the blocks were formed based on the related variables and half of the samples were considered as intervention and control group in each block. The main purpose of using this method is to create balances in the number of participants in each category (Kang, Ragan, & Park, 2008).

### 2.1 Instruments

Goldberg's General Mental Health Questionnaire is a 24‐item standard questionnaire widely used for general population and for the assessment of mental health to conduct screening in the primary care level. Four subscales in the psychopathology domains are available as follows:

Physical symptoms (items 1–7) focus on how people feel about their health status and their fatigue with physical symptoms:

1. Anxiety and sleep disorders symptoms (items 8–14) which are related to insomnia and anxiety.
2. Social function (items 15–21) is concerned with the people's ability to cope with the demands of professional and everyday life issues and reveals how people feel in coping with common situations of life.
3. Depression symptoms (items 22–28) measure acute depression and suicidal tendencies.

To analyse the results of the questionnaire, one score was given for each personal dimension, as well as for the total scores for each individual. In GHQ scoring method, the items A to D are given 0, 1, 2, 3 scores respectively. Therefore, each individual's score at any dimension ranges from 0–21 and in total 0–84. Then, the average scores in each dimensions of mental health were calculated. According to the obtained results from the studies of Noorbala, Bagheri Yazdi, and Mohammad (2008), Williams, Goldberg, and Mari (1987), Palahang et al. (1996) and Yaghoubi, Nasr, and Shahmohammadi (1995), the score of 23 was considered as the cut off for the questionnaire, by which sensitivity and specificity of the test were 70.5 and 92.3, respectively. The subjects whose scores were above 23 were considered as suspicious to disorder. At each dimension, a score of 6 and above and a total score of 24 and above is regarded as the symptoms of disease (Noorbala et al., 2008).

The questionnaire has been replicated in different studies and its validity and reliability has been confirmed. Ebrahimi, Molavi, Moosavi, Bornamanesh, and Yaghobi (2007) reported the criterion validity of the instrument as 0.78. Malakooti et al. (2006) calculated the reliability coefficient through Cronbach's alpha coefficient and test‐retest as 0.6.

### 2.2 Intervention

The intervention groups were treated by group integrative reminiscence therapy including 10 sessions, twice a week for 60–90 min with the help of the researcher, psychologist and co‐researcher. Given that integrative reminiscence group therapy focuses on the acceptance of one's past, attitudes towards one's past life, looking at older people age as a valuable and satisfying period, finding meaning in life and the integration of present and past, the acceptance of negative past events and recognizing a continuous pattern between past and present (Bohlmeijer, Kramer, Smit, Onrust, & van Marwijk, 2009), the following sessions were held for all subjects:

Session 1: Introducing oneself and a brief family history (who are the family members and what effects, whether positive or negative, they have had on one's life, defining the objectives of the group.

Session 2: Emphasizing great works and honors and highlighted roles of the individuals at home, family and community.

Session 3: Adopting decisive events in one's life (a decisive event is an experience one has had in his or her life leading to a major change in life.

Session 4: Talking about the childhood memories and favorite games.

Session 5: Recounting the memories related to adolescence and youth (love and hate).

Session 6: Talking about the memories of wooing and marriage.

Session 7: Focusing on memories related to children and grandchildren birth.

Session 8: Emphasizing the memories of the opposed war and its effects on their lives.

Session 9: Elaborating the meaning and purpose of life (We choose some goals in our lifetime which give us a sense of purpose).

Session 10: At the end of the sessions, with the help of psychologist and the researcher, the subjects evaluate and interpret the provided matters and try to find and make sense of events which they have been in contact with, accept the past negative experiences and integrate them with her current life.

At the beginning of the intervention, a series of activities such as singing part song or a simple health exercise to create a pleasant and favorable feeling were assigned for the intervention group. During the sessions, the participants were encouraged to review the important past events of their life and draw a conclusion. In addition, old photos and songs were used to stimulate and refresh the memories related to the past achievements and help older people to get the meaning of life. A short time was allocated to the participants’ refreshment between the sessions. The control group received no intervention and the typical programs for the nursing home were only used. After 1 month from the last group integrative reminiscence therapy session, Goldberg mental health questionnaire was given to both experimental and control group.

### 2.3 Data analysis

To analyse the data, descriptive statistics such as frequency and percentage and inferential statistics such as ANCOVA and Eta coefficient were used.

## 3 RESULTS

Based on the results, the majority of participants in both experimental and control groups (37%) were aged 84–75 years. Table 1 indicates the frequency and demographic variable percentage such as age, educational status, education, number of children, occupation and length of residency and the reason for staying at nursing home.

**Table 1:** The demographics in both intervention and control groups

Variable		Control group		Intervention group		Total	
		Frequency	Per cent	Frequency	Per cent	Frequency	Per cent
Age	74–60	7	30.4	6	26.1	13	28.3
	84–75	6	26.1	11	47.8	17	37
	Over 84 years	10	43.5	6	26.1	16	34.8
	Total in group	23	100	23	100	46	100
Marital status	Single	3	13	5	21.7	8	17.4
	Divorced	3	13	0	0	3	6.5
	Married	4	17.4	3	13	7	15.2
	Widow	13	56.5	15	62.2	28	60.9
	Total in group	23	100	23	100	46	100
Education	Illiterate	13	56.5	16	69.6	29	63
	Primary	7	30.4	6	26.1	13	28.3
	Intermediate	2	8.7	1	4.3	3	6.5
	Diploma	1	4.3	0	0	1	2.2
	Total in group	23	100	23	100	46	100
Number of children	No children	10	43.5	10	43.5	20	43.5
	3–1 children	8	34.8	6	26.1	14	30.4
	6–4 children	5	21.7	7	30.4	12	26.1
	Total in group	23	100	23	100	46	100
Job	Housewife	19	86.4	19	86.4	38	86.4
	Employee	0	0	1	0	1	2.2
	Self employee	1	4.3	0	4.3	1	2.2
	Unemployed	3	13	3	13	6	13.6
	Total in group	23	100	23	100	46	100
Duration of stay	Less than a year	6	26.1	7	30.4	13	28.3
	Between one and 5 years	17	73.9	16	69.6	33	71.7
	Total in group	23	100	23	100	46	100
Reason of referral	Loneliness	6	26.1	9	39.1	15	32.6
	Inability to care for themselves	14	60.9	13	56.5	27	58.7
	Lack of housing	2	8.7	0	0	2	4.3
	Lack of maintenance	1	4.3	1	4.3	2	4.3
	Total in group	23	100	23	100	46	100

As shown in Table 2, ANCOVA results indicated that integrative reminiscence group therapy could significantly influence on mental health with a confidence level of 0.95 (*F* = 82.450, *p *=* *.000). In addition, based on ETA coefficient, 0.65% of the variation is related to integrative reminiscence group therapy.

**Table 2:** Analysis of Covariance mental health factor by removing the effect of pre‐test

Statistical indicators	Sum of squares	Degrees of freedom	Mean square	*F*	Significance level	Eta coefficient
Pre‐exam	4759.909	1	4759.909	346.267	0.000	0.89
Group	1133.382	1	1133.382	82.450	0.000	0.675
Error	13.746	43	13.746			
Total corrected	6468.130	45				

Regarding the second hypothesis, as it is evident from Table 3, integrative reminiscence group therapy played a significant role on physical (*F* = 26.018; *p *<* *.001), anxiety and sleep disorder (*F* = 14.257; *p *<* *.001) and social disorder (*F* = 26.266; *p *<* *.001) and depression (*F* = 30.020; *p *<* *.001) dimension. Furthermore, ETA coefficient was 0.37 in the physical dimension, anxiety and sleep disturbances, 0.24 in social function dimension and 0.41 in depression dimension which is related to the effect of group integrative reminiscence therapy.

**Table 3:** Mental health Analysis of Covariance by removing the effect of pre‐test

Mental health dimensions	Statistical indicators	Sum of squares	Degrees of freedom	Mean square	*F*	Significance level	Eta coefficient
Physical	Pre‐exam	794.685	1	794.685	210.807	0.000	0.83
	Group	98.079	1	98.079	26.018	0.000	0.37
	Error	162.098	43	3.770			
	Total corrected	1161.326	45				
Anxiety and sleep disorders	Pre‐exam	628.832	1	628.832	229.077	0.000	0.842
	Group	72.101	1	72.101	26.266	0.000	0.379
	Error	118.038	43	2.745			
	Total corrected	803.413	45				
Social dysfunction	Pre‐exam	746.583	1	746.583	455.057	0.000	0.914
	Group	23.391	1	23.391	14.257	0.000	0.249
	Error	70.547	43	1.641			
	Total corrected	831.826	45				
Depression	Pre‐exam	896.908	1	896.908	259.299	0.000	0.858
	Group	103.838	1	103.838	30.020	0.000	0.411
	Error	148.736	43	3.495			
	Total corrected	1105.995	45				

## 4 DISCUSSION

The results of the present study indicated that group integrative reminiscence therapy had a positive and significant effect on mental health and its dimensions including physical, anxiety and insomnia, social dysfunction and depression dimension among older people in Dezful nursing home after a 10‐session intervention over 4 weeks. Choosing the integrative type of reminiscence including a review of the facts, past events, meaningful dimensions of life and its connection with the present as well as group intervention for the purpose of social and emotional support, stress reduction and social interactions among the older people could play a key role for the results. In another study, the older women were given priority on the basis of their vulnerability to stress and mental and physical pressures caused by ageing due to their specific socio‐economic conditions (Moradinezhad, Sahbaei, Nekavand, & Zarea, 2010).

The results of the present study were consistent with the study of Wu (2011), which integrative reminiscence group therapy resulted in increasing confidence and life satisfaction and reducing depressive symptoms. Bohlmeijer et al. (2009) indicated a positive impact of integrative reminiscence on reducing depression. In addition, the results are in line with the study of Sotodeh, Pooragha Roodbordeh, Kafi, and Poornesaii (2012), which proved a positive and increasing effect of reminiscence group therapy on mental health among older men. Furthermore, the results are congruent with some studies like Sahebdel, Khoshkonesh, and Pourebrahim (2012), Moradinezhad et al. (2010), Fakhar, Navabinejad, and Foroghan (2008) which improved mental health through group intervention and reminiscence. In another study, Huang et al. (2009) increased cognitive function scores after 8 sessions of reminiscence although the increase was not statistically significant. The difference in the results of these studies may be related to the kind of reminiscence and the duration of the intervention. In conclusion, group therapy played a significant impact on mental and physical health among older people living in nursing homes.

Group establishments provide precious experience and opportunity to establish a genuine and friendly connection, along with empathy to members, the opportunity which never happens in individual psychotherapy (Navvabinejad, 2004). Glasser (2006) believes that the sense of responsibility, controlling and responding to needs, especially the need for love and belonging which flourishes in the group can considerably affect the mental health.

Regarding the effectiveness of reminiscence, Erikson (1982) emphasized that the treatment can help the older people to review their past leading to pass the Integrity stage of character during despair. Considering the results of our study, it seems that holding group integrative reminiscence sessions in depressed environment of nursing home can play an important role in mental health. Thus, promoting each of the dimensions among the older women as a psychotherapy intervention through responding to their social, psychological and emotional needs is essential.

As for the physical dimension, the results indicated that physical health of older people could significantly increase, which are in line with the results of Hsu and Wang (2009). In Iran, some studies such as Sotodeh et al. (2012), Moradinezhad et al. (2010) and Nemati Dehkordi, Bozorgi, Pakseresht, and Rasekh (2007) also showed. However, the results are inconsistent with Wang, Yen, and OuYang's (2009) study, where no significant change was observed in promoting physical health after implementing reminiscence therapy. Furthermore, the results were incongruent with some studies like Sahebdel et al. (2012) and Fakhar et al. (2008). Regarding the main reason for inconsistency in the results, unsatisfactory physical health of older people living in nursing homes, dementia and its impact on the loss of physical function and the problems of old age can be emphasized (Sahebdel et al., 2012; Wang, 2005).

As for the positive role of anxiety and sleep disorder dimension, of the results were consistent with the studies of Zauszniewski (2004), Wang et al. (2009) and Moradinezhad et al. (2010). The fear of death was regarded as the most influential factor for fear and anxiety among older people. Fear and anxiety among older people is related to ageing, lost opportunities, aimless life and social isolation (Fakhar et al., 2008, pp;.59–60). If older people can participate in integrated group sessions and review past life dimensions and its meaning, as well as know their actual role in past events, they can reduce anxiety and have a better performance. In addition, satisfying the need to love and be loved during group meetings results in finding peace and achieving success (Glasser, 2006).

As far as the influential role of social function dimension, the results were congruent with the results of some studies like Nemati Dehkordi et al. (2007), Hsu and Wang (2009), Moradinezhad et al. (2010), Sotodeh et al. (2012). Group and group therapy can create a favorable and happy environment like home, reduce isolation and help making successful and emotional relationships with others as well as improving relations and social performance (Fakhar et al., 2008; Navvabinejad, 2004). Regarding depression, the present study indicated a significant decrease in depressive symptoms among older women which was inconsistent with the results of Chiang et al. (2009), Hsu and Wang (2009), Wang et al. (2009), Moradinezhad et al. (2010), Mohammadzaddeh et al. (2011) and Sotodeh et al. (2012). However, it contradicts with the results of Chao et al. (2006), Stinson and Kirt (2006) where significant change was not observed for improving depressive symptoms. The lack of significant results may be related to the small sample size, the inadequate number of meetings and having multiple health problems causing depression (Stinson & Kirt, 2006). Group integrative reminiscence therapy is similar to successful life review for older people living in nursing homes (Wu, 2011).

## 5 CONCLUSION

Based on the findings of the current study, group integrative reminiscence therapy resulted in improving and promoting mental health and its related dimensions among the older women living in nursing homes in Dezful, Iran.

The most important limitations lie in the lack of suitable physical space to hold meetings, the lack of proper cooperation of some older people with the researchers, inappropriate physical conditions of some older people, the illiteracy of the majority of the participants, the lack of a specific instrument to assess the mental health of older people and the low number of participants in research and lack of accessing to the older men.

In addition, some factors which may play a role in enhancing the favorability of nursing home environment, improving their mental health, and coping with the problems related to older people can be pinpointed in future studies such as briefing sessions for the nursing homes staff, introducing different types of psychotherapy, especially group therapy, reminiscence and its various methods, continuation of the group sessions, behaving correctly with older people and paying attention to their needs and demands. A comparative study between integrative reminiscence and other methods of group therapy is suggested for future studies.

## CONFLICT OF INTEREST

I have no conflicts of interest to declare.
